# hsCRP and ET-1 expressions in patients with no-reflow phenomenon after Percutaneous Coronary Intervention

**DOI:** 10.12669/pjms.334.13059

**Published:** 2017

**Authors:** Min Liu, Tian Liang, Peiying Zhang, Qing Zhang, Lei Lu, Zhongliang Wang

**Affiliations:** 1 Min Liu, Xuzhou City Hospital of TCM, Xuzhou 221009, Jiangsu Province, China, Nanjing University of Chinese Medicine, Nanjing 210029, Jiangsu Province, China; 2 Tian Liang, Xuzhou City Hospital of TCM, Xuzhou 221009, Jiangsu Province, China, Nanjing University of Chinese Medicine, Nanjing 210029, Jiangsu Province, China; 3 Peiying Zhang, Xuzhou Central Hospital, Xuzhou 221009, Jiangsu Province, China; 4 Qing Zhang, Nanjing University of Chinese Medicine, Nanjing 210029, Jiangsu Province, China; 5 Lei Lu, Nanjing University of Chinese Medicine, Nanjing 210029, Jiangsu Province, China; 6 Zhongliang Wang, Nanjing University of Chinese Medicine, Nanjing 210029, Jiangsu Province, China

**Keywords:** Coronary artery disease, ET-1, hs-CRP, Percutaneous coronary intervention

## Abstract

**Objective:**

To explore hsCRP and ET-1 expressions in patients with no-reflow phenomenon after percutaneous coronary intervention (PCI).

**Methods:**

A total of 136 patients with single coronary artery disease receiving PCI were divided into a reflow group and a no-reflow group to compare the level use of ET-1 alone with combined level of ET-1 and hs-CRP in PCI regarding sensitivity, specificity, positive and negative predictive values and accuracy for postoperative no-reflow. The study was conducted between 2014-2016 at our hospital.

**Results:**

Postoperative levels of ET-1 and hs-CRP in no-reflow group were significantly higher than those of reflow group (P<0.05). ET-1 level of reflow group peaked three hours after PCI and then declined. Serum level of hs-CRP decreased most obviously within three hours after PCI in reflow group and three hours - three days after PCI in no-reflow group. Left ventricular end-diastolic diameters of both groups after PCI were apparently lower than those before PCI, without significant inter-group difference (P>0.05). Left ventricular end-systolic diameters and left ventricular ejection fractions of both groups evidently increased after PCI, without significant inter-group differences either (P>0.05). Corrected TIMI frame count (CTFC) and wall motion score index of reflow group after PCI were significantly lower than those of no-reflow group (P<0.05). ET-1 level was positively correlated with CTFC (P<0.05). Multivariate linear regression showed hs-CRP was negatively correlated with the serum level (P<0.05) (r=-0.34).

**Conclusion:**

hsCRP and ET-1 levels significantly increased in patients with no-reflow phenomenon.

## INTRODUCTION

In general, acute myocardial infarction can be classified into acute ST-segment elevation myocardial infarction (STEMI) and acute non-ST-segment elevation myocardial infarction.^1^,^2^ For patients with STEMI, early rapid emergency thrombolytic therapy and/or percutaneous coronary intervention (PCI) can significantly reduce mortality and improve prognosis by clearing infarcted coronary arteries.^3^ However, thromboembolism, microthrombosis and other factors after PCI may cause no-recanalization of part of microvessels of the myocardium, resulting in no-reflow phenomenon and seriously affecting the clinical prognosis and outcomes of patients. No-reflow phenomenon means that part of infarct-related artery (IRA) has no forward blood flow after PCI in the absence of dissection, thrombosis, spasm or distal embolization.^4^

The pathophysiological mechanism of no-reflow remains unclear hitherto, but it has been attributed to severe local vascular spasm, obstruction, ischemia of corresponding tissues and organs (generally for 40 to 60 minutes), and release of many inflammatory factors. On this basis, no-reflow phenomenon easily occurs when the ischemic region cannot immediately be perfused with sufficient blood after recanalization for blood flow recovery.^5^

Endothelin (ET), which exists commonly in vascular endothelial cells and also widely in various mammalian tissues and cells, is an important factor regulating cardiovascular function. It plays a crucial role in maintaining basic vascular tension and cardiovascular system homeostasis.^6^ Endothelial cells are stimulated to synthesize and to release ET-1, mainly at the gene transcription level. ET-1 synthesis can be stimulated by epinephrine, thromboxane, vasopressin, angiotensin, insulin, cytokines and physical factors such as changes of vascular wall shear force and pressure and hypoxia. The process of stimulating ET-1 synthesis involves Ca^2+^-dependent protein kinases.^7^ The factors that inhibit ET-1 synthesis include NO, PGI2, atrial natriuretic peptide, heparin, etc. With a very short half-life in plasma (<5 min), ET-1 can quickly bind tissue receptors. It can be rapidly decomposed by ET-degrading enzymes, mainly in lungs and kidneys.^8^

High-sensitivity C-reactive protein (hs-CRP) has an extremely low content in the peripheral blood under normal conditions, but upon inflammation, such level can, as a stress reaction, markedly increase within a short time. It is well-documented that hs-CRP strongly indicate myocardial infarction and other inflammatory diseases.^9^ CRP is often synthesized by liver cells due to stimulation of interleukin-6 and other inflammatory molecules.^10^ In this study, we combined hs-CRP with ET-1 to detect their dynamic changes before and after PCI and the correlation, aiming to provide a scientific basis for the no-reflow phenomenon after PCI.

## METHODS

A total of 136 patients with single coronary artery disease admitted in our hospital from June 2014 to August 2016 were selected, including 78 males and 58 females aged 42-79 years old, (59.4 ± 18.7) on average. The patients after PCI were then divided into a reflow group (98 cases) and a no-reflow group (38 cases).

### Inclusion criteria

All enrolled patients which met the STEMI diagnostic criteria developed by the American College of Cardiology/American Heart Association.^2^ They suffered from various degrees of typical precordial pain or discomfort, finally diagnosed as single coronary artery disease by coronary angiography. This study was been approved by the ethics committee of our hospital. The patients and their families had signed informed consent.

***Exclusion criteria***

1)Complicated with other cardiovascular diseases such as endocarditis, valvular lesions and congestive heart failure;
2)Use of immunosuppressive agents;
3)Acute and chronic bacterial and/or viral infections;
4)Autoimmune diseases;
5)Connective tissue diseases;
6)Malignant tumors;
7)Liver and kidney dysfunctions;
8)Chronic muscular diseases;
9)Atorvastatin allergy;
10)Peripheral vascular diseases, chronic heart failure, thyroid diseases and surgeries within the last six months owing to major injuries;
11)Myocardial infarction, percutaneous transluminal coronary angioplasty or coronary artery bypass grafting within the last six months, recent use of adrenal cortex hormones or other immunomodulators, incompliance of patients or their families, and history of mental illness.

### PCI method

The IRA lesion was sucked 3-5 times with a thrombus suction catheter, which was increased when necessary to lower the thrombus load effectively and to open the forward blood flow. Afterwards, most of intracoronary thrombosis was eliminated to wipe out continuous retention of contrast agent, floating thrombus or residual of IRA distal thrombus fragments. After 50-100 μg nitroglycerin was intracoronarily injected, delayed angiography was performed. According to the vascular diameter, stent implantation was started when the stent successfully passed or the residual stenosis was less than 70%. Alternatively, pre-dilation was conducted before implantation at an appropriate pressure by using a balloon (5-14 atm). Prior to stent implantation or balloon pre-dilation, the contrast agent amount and the angiography number were minimized, and the interval between two angiographies was extended.

Method for determining immediate postoperative perfusion of epicardial blood vessels and myocardium: Two experienced interventional surgeons analyzed the TIMI blood flow grade, corrected TIMI frame count (CTFC) and wall motion score index (WMSI) of IRA.

### Criteria for successful treatment

In at least two orthogonal projections, angiography showed that the stent well adhered to the vascular wall and the residual stenosis was <20%, with TIMI grade 3 and without severe clinical complications such as sudden cardiac death, myocardial infarction and acute coronary artery bypass.

### Angiographic criteria for no-reflow phenomenon

Angiography disclosed no or obviously decelerated forward blood flow after PCI, with TIMI grade 2.

### Serological detection

Fasting cubital venous blood (8-12 ml) was drawn, of which 2 ml was used to detect hs-CRP levels before as well as half an hour, three hours and three days after PCI by rapid fluorescence immunoassay (Triage meter), and the remaining 4-6 ml was centrifuged at 1,200 r/minutes for 10 minutes. The resulting serum was stored in a refrigerator at -80°C, and the level of ET-1 was detected before as well as half an hour, three hours and three days after PCI by the enzyme-linked immunosorbent assay.

### Echocardiographic examination

Echocardiography was performed by two experienced ultrasound physicians strictly according to the guideline of American Society of Echocardiography. All patients received echocardiography by color Doppler diagnosis using a Philips iE33 ultrasound machine (probe frequency: 2.5 MHz) before PCI, on the first day after PCI and during follow-up respectively. The left ventricular end-diastolic diameter (LVEDD), left ventricular end-systolic diameter (LVESD) and left ventricular ejection fraction (LVEF) were recorded for statistical analysis.

### Statistical analysis

All data were analyzed by SPSS19.0. The qualitative data were compared by the χ^2^ test, and the ineligible fourfold table data were subjected to the Fisher’s exact test. The quantitative data were compared by the analysis of variance. All detected factors were subjected to the Pearson’s correlation analysis. P<0.05 was considered statistically significant.

## RESULTS

### Baseline clinical data

The reflow and no-reflow groups had similar gender ratio, age, fasting blood glucose level, LDL, HDL, BMI and MAP (P>0.05) (Table-I).

**Table-I:** Baseline clinical data.

*Index*	*Gender ratio (female/male)*	*Age*	*MAP (mmHg)*	*LVEDD (mm)*	*LVEF*	*LDL (U/L)*	*HDL (U/L)*	*FBG (mmol/L)*	*BMI (kg/m^2^)*
Reflow group	44/54	56.9±13.7	91.3±18.6	58.3±5.9	45.8±4.1	2.7±1.3	1.2±0.9	5.8±0.8	20.8±1.2
No-reflow group	21/37	55.5±18.7	92.4±27.5	58.7±4.5	44.4±4.5	2.7±0.9	1.4±0.8	5.4±1.3	20.4±1.8
T value	0.02	0.35	0.16	0.59	0.69	0.24	0.39	0.63	1.29
P value	0.34	0.25	0.28	0.42	0.41	0.44	0.69	0.29	0.12

### Postoperative biochemical indices

The postoperative levels of ET-1 and hs-CRP in the peripheral blood of no-reflow group were both significantly higher than those of the reflow group (P<0.05) (Table-II). The ET-1 level of the reflow group peaked three hours after PCI and then declined. The serum level of hs-CRP decreased most obviously within three hours after PCI in the reflow group and three hours-three days after PCI in the no-reflow group.

**Table-II:** Biochemical indices before and after PCI (χ̅±s).

*Index*	*Group*	*Case No.*	*0.5 h before*	*0.5 h after*	*three hours after*	*three days after*	*F*	*P*
ET-1 (mg/L)	Reflow group	98	128.8±22.8	71.3±18.4	57.2±16.8	52.3±12.7	4.18	0.01
	No-reflow group	38	159.4±69.5	99.4±15.9	91.7±31.5	91.3±32.4	0.19	0.31
	T value	-	0.76	20.37	27.3	31.4	-	-
	P value		0.45	0.01	0.02	0.01	-	-
hs-CRP (mg/L)	Reflow group	98	39.7±14.68	42.8±11.9	22.3±2.15	11.4±0.83	6.88	0.01
	No-reflow group	38	41.2±10.65	44.2±10.8	43.2±1.65	23.8±1.24	0.48	0.41
	T value	-	0.82	0.39	26.4	19.8	-	-
	P value		0.43	0.62	0.02	0.03	-	-

### Postoperative echocardiographic indices

LVEDDs of the two groups after PCI were apparently lower than those before PCI, but there was no significant inter-group difference (P>0.05). LVESDs and LVEFs of both groups evidently increased after PCI compared with those before PCI, without significant inter-group difference either (P>0.05) (Table-III).

**Table-III:** Postoperative echocardiographic indices (χ̅±s).

*Index*	*Group*	*Case No.*	*Before PCI*	*After PCI*	*T*	*P*
LVEDD (mm)	Reflow group	98	59.3±15.3	52.2±2.7	11.8	0.003
	No-reflow group	38	57.7±13.5	51.0±1.5	12.8	0.002
	T value	-	0.55	0.52	-	-
	P value		0.46	0.25	-	-
LVESD (mm)	Reflow group	98	42.1±4.7	39.2±1.3	13.2	0.002
	No-reflow group	38	41.8±5.2	38.6±1.8	9.4	0.021
	T value	-	0.78	0.46	-	-
	P value		0.32	0.63	-	-
LVEF (%)	Reflow group	98	45.8±5.1	45.2±4.6	11.4	0.021
	No-reflow group	38	42.9±5.5	44.3±4.3	8.2	0.032
	T value	-	0.68	0.49	-	-
	P value		0.51	0.38	-	-

### Immediate postoperative perfusion of epicardial blood vessels and myocardium

CTFC and WMSI of the reflow group after PCI were significantly lower than those of the no-reflow group (P<0.05) (Table-IV).

**Table-IV:** Immediate postoperative perfusion of epicardial blood vessels and myocardium.

*Index*	*Group*	*Case No.*	*Before PCI*	*After PCI*	*T*	*P*
CTFC	Reflow group	98	39.3±4.9	27.1±1.4	16.9	0.002
	No-reflow group	38	58.7±2.7	42.6±1.6	10.3	0.017
	F value	-	0.68	3.62	-	-
	P value		0.55	0.02	-	-
WMSI (mm)	Reflow group	98	42.1±3.9	38.2±1.7	18.2	0.001
	No-reflow group	38	42.8±5.8	39.6±1.8	9.3	0.021
	F value	-	0.22	3.98	-	-
	P value		0.76	0.02	-	-

### Correlations between serum ET-1 level and other factors

The Pearson’s correlation analysis showed that ET-1 level was positively correlated with CTFC in the patients (P<0.05) (Table-V). Multivariate linear regression analysis was performed with ET-1 level as the dependent variable and gender, age, mean arterial pressure, CTFC and WMSI as independent variables. Serum hs-CRP level was negatively correlated with ET-1 level (P<0.05) (Table-VI), with a correlation coefficient r of -0.34.

**Table-V:** Correlations between serum ET-1 level and other factors (r).

*Index*	*Gender*	*Age*	*hs-CRP*	*MAP*	*LVEDD*	*LDL*	*HDL*	*CTFC*	*FBG*	*BMI*	*LVEF*	*WMSI*
ET-1												
r	0.33	0.45	-0.34	0.36	0.48	0.54	0.87	0.26	0.18	0.34	0.47	0.51
P	0.34	0.25	0.02	0.28	0.31	0.44	0.29	0.04	0.39	0.38	0.42	0.01

**Table-VI:** Multivariate linear regression analysis for correlations between ET-1 and clinical indices.

*Variable*	*β*	*SE*	*β’*	*t*	*P*	*(95%CI)*	

						*Upper limit*	*Lower limit*
hs-CRP	0.531	0.143	0.784	0.432	0.02	0.16	0.67
WMSI	0.381	0.109	0.692	0.692	0.28	0.48	0.79

## DISCUSSION

No-reflow phenomenon was first found in the canine experiment. Animal experiments have shown that in the local myocardial ischemia model caused by ligation of canine coronary artery, the ligated artery is reopened for blood flow, when the ischemic area cannot be fully perfused.^11^ At present, no-reflow is a phenomenon that in case of severe spasm and obstruction in local blood vessels, the corresponding tissues and organs appear ischemic (40-60 min generally), when if the blood vessels are recanalized to restore blood flow, but the ischemic area cannot get sufficient blood perfusion.^12^ No-reflow phenomenon is commonly seen in the myocardium, but can also be found in the brain, kidneys, skeletal muscles, etc. No-reflow is actually caused by the duration of ischemic time of tissue damage and the superimposition of extent.^13^ The main reason for no-reflow is swelling of microvascular endothelial cells, increased interstitial pressure due to exudate in extravascular microvascular extracellular matrix, and clogging of microvessels caused by platelet aggregation and/or leukocyte impaction.^14^ In the course of no-reflow development, biological indicators such as endothelin and C-reactive protein play a crucial regulatory role.^15^

ET is an important factor in regulating cardiovascular function, which plays a key role in maintaining basal vascular tension and the stability of cardiovascular system.^16^ ET is a polypeptide composed of 21 amino acids, with a molecular weight of 2400 D. Its N-terminus is two disulfide bonds linking the cysteine in sites 1-15 and 3-11, and its C-terminus is some hydrophobic amino acid residues. The N-terminal structure determines its affinity with the receptor, and the C-terminal structure the binding site with the receptor.^17^ ET-1 also has two isomeric families, i.e. ET-2 and ET-3, which differ in the residues of individual amino acids. ET-1 plays a major role in the cardiovascular system.^18^ In coronary atherosclerotic heart diseases, coronary endothelial cells are stimulated to synthesize and release ET-1, whose regulation is at the level of genetic transcription. Factors that stimulate ET-1 synthesis include epinephrine, thromboxane, vasopressin, angiotensin, insulin, and cytokines, as well as physical factors such as changes of vascular wall shear stress and pressure, and anoxia. And the stimulating process of ET-1 synthesis needs the involvement of Ca^2+^ (dependent protein kinase C).^19^ Factors that inhibit ET-1 synthesis include NO, PGI2, atrial natriuretic peptide, heparin, etc. ET-1 half-life in plasma is very short (<5 minutes), which can quickly combine with tissue receptors and decomposed by ET-degrading enzymes, with its sites of clearance mainly in the lungs and kidneys.^20^ Therefore, ET-1 can be used as one of the serological markers of CHD.^21^ In addition, the Centers for Disease Control and Prevention and the American Heart Association (suggest that cardiovascular risks of patients can be categorized according to hs-CRP levels: <1 mg/L for relatively low risk, 1.0 to 3.0 mg/L for moderate risk, >3.0 mg/L for high risk.^22^ The two indicators are clinically important for evaluating no-reflow in myocardial infarction, but their relevance remains unclear.^23^

In our study, the levels of ET-1 and hs-CRP in peripheral blood after PCI in the no-reflow group were significantly higher than those in the reflow group, with the difference statistically significant (P<0.05). The ET-1 level in the reflow group peaked at three hours after PCI and then declined. The level of ET-1 did not show any significant change in both the reflow group and the no-reflow group (P>0.05). The level of serum hs-CRP had the largest amplitude of decrease within three hours after PCI in the reflow group and at three hours-three days after PCI in the no-reflow group respectively. The results were consistent with those of Ding et al.^24^ LVEDDs of the two groups after PCI were significantly lower than those before PCI, between which, however, there was no significant difference (P>0.05). LVESDs and LVEFs of the two groups after PCI were significantly increased, with the differences not statistically significant (P>0.05), suggesting that whether there is reflow or not has no significant short-term impact on cardiac remodeling. We believe that this may be due to the reason that in addition to increased left ventricular end-diastolic diameter and increases ejection fraction caused by impaired ischemic myocardial contractility, cardiac remodeling can also be regulated by multiple neuroendocrine factors, such as vasopressin and atrial natriuretic peptide. CTFC and WMSI of the reflow group after PCI were significantly lower than those in the no-reflow group, with the differences being statistically significant (P<0.05). The Pearson’s correlation analysis showed that the ET-1 level was positively correlated with CTFC in STEMI patients (P<0.05). The multiple linear regression analysis was made with ET-1 level as the dependent variable, gender, age, MAP, CTF and WMSI as independent variables, and the results showed that hs-CRP might be a factor influencing the serum level of STEMI patients (P<0.05), between which there was a negative correlation, with a correlation coefficient of r=-0.34. These results suggested that the combined monitoring of ET-1 and hs-CRP levels after PCI may have important clinical value for indicating postoperative reflow.

## CONCLUSION

In conclusion, hsCRP and ET-1 levels significantly increased in patients with no-reflow phenomenon.

### Author’s Contribution

***ML, TL, QZ, LL & ZW:*** Data collection and analysis.

***PZ:*** Study design and manuscript writing.
